# Linking Inflammation to Reduced Food Intake in Advanced Cancer: A Prospective Observational Study

**DOI:** 10.3390/curroncol33040209

**Published:** 2026-04-08

**Authors:** Asta Bye, Trude Rakel Balstad, Ida Ervik Raaness, Tora Skeidsvoll Solheim, Ragnhild Habberstad, Pål Klepstad, Erik Torbjørn Løhre, Olav Faisal Dajani, Stein Kaasa, Nina Aass, Ola Magne Vagnildhaug

**Affiliations:** 1 Department of Nursing and Health Promotion, Faculty of Health Sciences, OsloMet—Oslo Metropolitan University, 0130 Oslo, Norway; 2 European Palliative Care Research Centre (PRC), Department of Oncology, Oslo University Hospital, and Institute of Clinical Medicine, University of Oslo, 0424 Oslo, Norwaynaa@ous-hf.no (N.A.); 3 Department of Clinical and Molecular Medicine, Faculty of Medicine and Health Sciences, NTNU—Norwegian University of Science and Technology, 7491 Trondheim, Norway; trude.r.balstad@ntnu.no (T.R.B.);; 4 Department of Clinical Medicine, Clinical Nutrition Research Group, UiT The Arctic University of Norway, 9019 Tromso, Norway; 5 Department of Clinical Medicine, Faculty of Medicine and Dentistry, University of Bergen, 5009 Bergen, Norway; idraan@ous-hf.no; 6 Cancer Clinic, St. Olavs Hospital-Trondheim University Hospital, 7006 Trondheim, Norway; 7 Department of Circulation and Medical Imaging, NTNU—Norwegian University of Science and Technology, 7491 Trondheim, Norway; 8 Department of Anesthesiology and Intensive Care Medicine, St. Olavs Hospital-Trondheim University Hospital, 7006 Trondheim, Norway; 9 Centre for Crisis Psychology, Faculty of Psychology, University of Bergen, 5009 Bergen, Norway; 10 Regional Advisory Unit for Palliative Care, Department of Oncology, Oslo University Hospital, University of Oslo, 0318 Oslo, Norway

**Keywords:** cancer, food intake, inflammation, CRP, PG-SGA, 24 h dietary recall, weight loss

## Abstract

**Background**: Undernutrition and cachexia are common in advanced cancer and often linked to systemic inflammation. While inflammation is associated with poorer prognosis, accelerated weight loss, and reduced treatment tolerance, its direct impact on food intake remains insufficiently investigated. **Aim**: To examine the association between systemic inflammation and energy and protein intake over time in patients with advanced cancer. **Methods**: A total of 170 patients from the Palliative Radiotherapy and Inflammation Study were included. Nutritional status was assessed using PG-SGA SF. Dietary intake was recorded using repeated 24 h recalls. Systemic inflammation was defined as CRP > 10 mg/L. Mixed linear models were applied to evaluate the association between inflammation energy and protein intake over time. **Results**: Systemic inflammation (CRP >10 mg/L) was present in 87 (51%) patients and associated with significantly lower energy (−3.6 kcal/kg, *p* = 0.04) and lower protein intake (−0.25 g/kg, *p* = 0.003). Patients with inflammation were more often undernourished and had shorter survival. **Conclusions**: Systemic inflammation is likely associated with clinically relevant reductions in energy and protein intake in advanced cancer. CRP may help identify patients for whom standard nutritional support is insufficient.

## Simple Summary

Many people with advanced cancer find it hard to eat enough. One reason for this may be inflammation, a reaction in the body that can increase as cancer progresses. In this study, we followed 170 patients receiving palliative radiotherapy and asked them what they ate over several days. We also measured inflammation using a simple blood test (CRP). We found that patients with higher inflammation ate less than those with lower inflammation. They were also more likely to be undernourished and live for a shorter time. Although patients with higher inflammation ate less, we cannot tell from this study whether this is due to appetite or other factors. Keeping track of CRP levels may help healthcare professionals recognize patients who could benefit from extra support.

## 1. Introduction

In cancer, undernutrition is a frequent complication affecting 20–85% of the patients, reducing both quality of life and prognosis. Additionally, up to 50–80% of patients with advanced disease develop cancer cachexia, a catabolic syndrome characterized by disproportionate skeletal muscle loss [1,2,3,4].

Undernutrition is primarily driven by inadequate energy and protein intake [5]. It triggers adaptive physiological responses, including reduced energy expenditure and a metabolic shift toward fat utilization in order to preserve muscle mass [6]. Appetite loss is a major contributor [2], often intensified by other nutrition impact symptoms [2,7,8]. In contrast, cachexia represents a distinct catabolic state in which systemic inflammation disrupts energy and protein metabolism, leading to progressive loss of body mass with a substantial reduction in skeletal muscle [6,9,10]. Furthermore, and in contrast to undernutrition, cachexia is characterized by elevated resting energy expenditure, heart rate, and body temperature, which accelerate muscle protein breakdown to support gluconeogenesis and acute-phase protein synthesis [6]. Consequently, muscle loss persists even in those with adequate intake [5].

Although systemic inflammation is a defining feature of cachexia, its impact on food intake remains unclear. This distinction is clinically important, as nutritional support alone may be insufficient if inflammation suppresses appetite [11]. Existing studies have suggested that inflammation may directly suppress dietary intake [12,13,14]. However, the studies mainly rely on crude measures, such as single self-reported questions about eating less than usual [12,13] rather than validated dietary assessments.

C-reactive protein (CRP) is widely used as a marker for systemic inflammation, rising in both acute and chronic conditions [15]. CRP is incorporated in both the Global Leadership Initiative in Malnutrition (GLIM) criteria [8] and the modified Glasgow Prognostic Score (mGPS) [16]. Our previous studies show that systemic inflammation predicts both short-term and long-term weight loss and is associated with appetite loss [17]. However, whether elevated CRP is associated with lower measured energy and protein intake is unclear. We therefore tested the hypothesis that systemic inflammation, measured by CRP, is inversely associated with directly measured energy and protein intake in patients with advanced cancer.

## 2. Methods

### 2.1. Patients and Study Design

This study utilized data from the Palliative Radiotherapy and Inflammation Study (PRAIS), a multicenter longitudinal observational study conducted across seven European centers between 2013 and 2017 [18]. Key eligibility criteria were established cancer diagnosis, referral to palliative radiotherapy for verified (CT/MRI) painful bone metastasis, age 18 or older, and the ability to comply with trial procedures. Patients were not included if they had ongoing radiotherapy, had received radiotherapy within the previous four weeks, or had a pathological fracture of a long bone.

Of the 574 patients enrolled in PRAIS, 180 patients were recruited from Oslo University Hospital between January 2015 and December 2017. These patients were asked to provide dietary information. After applying the eligibility criterion of available baseline CRP measurements, 170 patients were included in the present analysis.

### 2.2. Data Collection

Data were collected at three timepoints: before radiotherapy, and three and eight weeks after radiotherapy. Demographic details (age, gender, living situation, length of education), primary diagnosis, height, weight and date of death were obtained from the case report forms. Body weight was measured at every study visit, and percentage change was calculated from baseline to week eight. The Patient-Generated Global Assessment short form (PG-SGA SF) [19,20] was used as a screening tool for risk of malnutrition. A score between 2 and 8 indicates a moderate risk of malnutrition, while a score >9 indicates a severe risk, which requires further assessment [21]. Data on appetite was collected by the EORTC QLQ-C15 PAL [22], which includes the following item: During the past week, have you lacked appetite?, rated on a four-point scale from not at all to very much.

Standardized 24 h recall interviews were completed to collect data on food intake, following established methodology [23,24]. A trained nurse interviewed patients face-to-face, asking them to recall all foods and beverages consumed during the previous day, from midnight to midnight. A dietitian was available to provide advice and guidance when needed. Portion sizes were estimated using household measures from a photographic booklet [25]. These included common items such as cups, glasses, spoons, slices, bowls, and pieces. These visual aids were then translated into gram weights for nutrient analysis. The dietary data were processed using the software package Avio 2000 (SVIO AB, Stockholm, Sweden). Nutrient intake was calculated based on the Norwegian food composition tables [20], supplemented with locally developed recipes and brand-specific product information. From this, estimates of total energy intake (kcal/day) and macronutrient intake (protein, fat, carbohydrates, alcohol) (g/day) were derived for each patient. For the present analysis, energy and protein intake per kilogram of body weight were calculated and applied.

Clinical chemistry analyses, including CRP, were performed at the local laboratory facility. CRP above 10 mg/L was used to indicate clinically significant systemic inflammation. This cutoff is identical to the one applied in the mGPS, which has been extensively validated [16]. Although dichotomizing a continuous variable inevitably reduces the granularity of the data, it offers the advantages of simpler interpretation and ensures that the assumptions underlying linear regression are met.

### 2.3. Statistical Analysis

Descriptive baseline characteristics were presented both overall and stratified by CRP levels. Continuous variables were summarized as means with standard deviations (SD) or medians with interquartile ranges (IQR: 25th–75th percentiles). Categorical variables were reported as percentages. Linear regression was used to test for differences between groups in continuous variables, and a chi-square test was used to test for differences in categorical variables. To estimate the effect of systemic inflammation on energy and protein intake over time, mixed linear modeling for repeated measurements was performed using a random intercept model, adjusting for potential confounders. A random intercept model was chosen over a random slope model after a sensitivity analysis showed that model parameters were comparable between the two. Time was categorized according to the three study visits (baseline and 3 and 8 weeks after the end of radiotherapy). The following possible confounders were chosen among baseline characteristics based on previous knowledge of associations with both the predictor (CRP) and the outcome (protein or energy intake): age, sex, performance status, primary tumor type and systemic anti-cancer treatment. These covariates were added to the model by forced entry. Data were assumed to be missing at random (MAR), meaning that missingness depends on observable data in the dataset. Mixed linear modeling handles this very well, and the need for listwise deletion or other strategies for handling missing data was not thought to be required. The level of statistical significance was set to a *p*-value < 0.05. Stata MP version 18.0 (College Station, TX, USA) was used for statistical analysis.

### 2.4. Ethics

The PRAIS was approved by the Regional Committee for Medical and Health Research Ethics (REC Protocol Approval 2013/1126) [18]. All patients provided written informed consent before inclusion. The study was carried out in accordance with ICH GCP and the World Medical Association Declaration of Helsinki (1964). All data were handled anonymously; patients were identified by an assigned code.

## 3. Results

A total of 170 patients were included in this analysis. Table 1 summarizes the baseline characteristics stratified by systemic inflammation (CRP < 10 mg/L (*n* = 83, 48.8%) vs. CRP > 10 mg/L (*n* = 87, 51.2%)). One hundred (59%) were male (Table 1) and the median age was 65 (IQR 15) years. The most common diagnoses were gastrointestinal (26%, *n* = 45), prostate (22%, *n* = 38) and breast cancer (21%, *n* = 35). At baseline, 78% of patients (*n* = 126) were at risk of undernourishment, with 48% moderately and 30% severely undernourished.

The two groups were comparable in terms of age, sex, and living situation. Breast cancer was less prevalent among patients with CRP > 10 mg/L (10% vs. 31%). Patients with CRP > 10 mg/L were significantly more likely to be undernourished (*p* = 0.002). Severe undernutrition occurred in 39% of these patients compared to 19% in those with low CRP. Although not statistically significant, patients with elevated CRP tended to experience greater weight loss (mean 4.6% vs. 2.4%, *p* = 0.077) and reported more frequent appetite loss.

Table 2 presents energy and protein intake per kg body weight, as well as weight loss over time, stratified by CRP group. Mixed linear model analysis, including all patients with data at ≥1 time point, showed that patients with systemic inflammation had significantly lower energy (−3.6 kcal/kg, SE 1.7, *p* = 0.038) and protein intake (−0.25 g/kg, SE 0.083, *p* = 0.003) over time compared to patients without systemic inflammation (Table 3). These differences remained constant throughout the study period with no significant interaction with time, and were independent of potential confounders (age, sex, performance status, primary tumor type, systemic anti-cancer treatment).

## 4. Discussion

This observational study found that systemic inflammation was independently associated with significantly lower energy (−3.6 kcal/kg) and protein (−0.25 g/kg) intake after adjusting for key covariates (age, sex, physical performance status, primary tumor type, and systemic cancer treatment). For a 70 kg individual with inflammation, this represents a daily deficit of ~250 kcal and ~17 g of protein, which is clinically relevant with respect to weight loss in a population where 86% of the patients were classified at risk to be undernourished at baseline [26].

Our findings indicate that systemic inflammation is independently associated with reduced food intake in patients with advanced cancer, consistent with the central role of inflammation in cachexia [6]. This aligns with previous research linking elevated CRP to reduced food intake [12,13,14]. Importantly, only one of these studies [14] directly measured food intake in cancer patients receiving palliative care. Similarly, a large European study in geriatric patients identified CRP ≥ 3.0 mg/dL as the strongest predictor of low food intake, surpassing age, infection, and comorbidities [13]. Together, these findings indicate that it is important to consider systemic inflammation when identifying the risk of malnutrition. However, they do not necessarily imply that nutritional support is effective in patients with inflammation. Merker et al. [27] examined whether inflammatory status influenced the effectiveness of nutritional support on mortality and found no significant benefit among patients with high inflammation (CRP > 100 mg/L). The authors suggested that inflammatory status at admission may guide individualized nutritional strategies, potentially with lower intake targets for those with markedly elevated inflammation.

CRP is an acute-phase protein produced by the liver in response to pro-inflammatory cytokines, such as interleukin-6 (IL-6), tumor necrosis factor (TNF), and interleukin-1 beta (IL-1β) [28,29]. These cytokines are presumed to disrupt central appetite regulation and alter metabolism, suppressing appetite-regulating hormones like ghrelin, leading to appetite loss and reduced energy and protein intake [30]. Inflammation, particularly driven by IL-6 and TNF, also exacerbates weight loss by increasing resting energy expenditure and causing metabolic disturbances [11]. In addition, growth differentiation factor 15 (GDF-15), a stress- and inflammation-induced cytokine associated with cancer cachexia has been shown to suppress appetite and contribute to metabolic alterations [31]. Collectively, these mechanisms illustrate the multifaceted ways in which systemic inflammation may affect nutritional status in patients with advanced cancer. Findings from studies on cancer cachexia anorexia [31] and a recent review on cachexia models [32] further support that inflammatory mediators such as IL-1, IL-6 and GDF-15 play central roles in appetite suppression and metabolic disturbances. Inflammation, therefore, appears to influence both appetite and metabolism, underscoring the need for integrated approaches that address inflammatory pathways in this vulnerable population. However, the optimal anti-inflammatory treatment for cancer cachexia remains unclear [31]. Potential approaches include targeted agents such as ponsegromab (a GDF-15 inhibitor), broader anti-inflammatory options like NSAIDs, and cytokine-directed therapies such as IL-6 pathway inhibitors, although other emerging treatments may also prove relevant as evidence evolves.

The observed association between systemic inflammation and reduced energy and protein intake in the present study is relevant to the pathophysiology of cancer cachexia [33]. Persistent low energy intake in patients with inflammation, even after adjusting for confounders, is compatible with an association between inflammation and negative energy balance in cachexia. In the present study, risk of malnutrition was assessed by using PG-SGA SF. Among patients with inflammation, a significantly higher proportion were identified as at high risk of malnutrition compared to those with low CRP. This association raises the question of whether these patients were primarily undernourished or experiencing cancer cachexia [34]. It should also be acknowledged that, given the observational design, a reverse association between inflammation and reduced intake cannot be excluded. Inadequate energy and protein intake may contribute to metabolic stress through catabolism and tissue loss. However, findings from cardiovascular populations indicate that undernutrition may influence inflammatory physiology without increasing CRP, likely because poor nutritional status can attenuate the hepatic synthesis of acute-phase proteins [35].

PG-SGA SF is a widely used screening tool in clinical practice, but it emphasizes intake- and symptom-related items and provides no information on inflammatory burden. In advanced cancer, this may hinder differentiation between undernutrition primarily driven by low intake and cachexia, in which systemic inflammation plays a central role. According to current guidelines [33], this distinction is crucial for guiding appropriate treatment since nutritional interventions are regarded as insufficient in the presence of ongoing systemic inflammation [26]. Our findings support the use of the GLIM criteria, which specify inflammation as a key component of the diagnosis of cancer cachexia [36]. Consistent with the guidelines, a multimodal approach that combines anti-inflammatory strategies with individualized nutritional support may be necessary to address cachexia effectively and improve patient outcomes [33]. Future research should therefore focus on interventions that target both inflammation and nutrition, as well as the development of practical clinical tools that incorporate inflammatory status to better distinguish between undernutrition and cachexia.

Another finding that underscores the importance of evaluating inflammation when identifying undernutrition in patients with advanced cancer is the tumor-specific variation observed in our cohort. Breast cancer patients were notably underrepresented among those with systemic inflammation. This aligns with clinical experience, where patients with breast cancer, even in advanced stages, less frequently develop undernutrition and cachexia compared to those with gastrointestinal or lung cancer [37]. The lower prevalence of elevated CRP in this subgroup may reflect distinct tumor biology, characterized by less pronounced systemic inflammatory response despite disease progression [38]. Consequently, nutritional decline in breast cancer patients may be more closely linked to intake-related factors rather than inflammation-driven metabolic alterations.

## 5. Strengths and Limitations

A strength of this study is its longitudinal design, which enabled the assessment of changes in energy and protein intake over time in relation to systemic inflammation. The use of repeated 24 h dietary recalls provided direct and detailed information on actual food intake, which is considered a reliable method at the group level in clinical nutrition research [39,40]. Additionally, the inclusion of CRP as an objective biomarker of inflammation strengthens the study’s ability to explore the relationship between inflammation and nutritional intake [41].

However, several limitations should be acknowledged. Given the observational design, the study cannot establish causal relationships between systemic inflammation and nutritional intake. While 24 h recalls are widely used, they are subject to recall bias and may not fully capture individual habitual intake [39,40]. However, our aim was to describe energy intake at the group level at each measurement point, for which single recalls are considered acceptable. The study population was mainly composed of patients with metastatic gastrointestinal, prostate, and breast cancer, which may limit generalizability to other cancer types and stages. In addition, as this analysis includes only the Oslo University Hospital subset of the PRAIS cohort, the findings may be subject to selection bias and should be interpreted with caution regarding generalizability.

Furthermore, although CRP is a well-established marker of systemic inflammation, it is nonspecific and may be elevated due to factors unrelated to cancer or malnutrition, reflecting cytokine-driven acute-phase activation rather than disease-specific processes [41,42]. Thus, a single elevated CRP measurement may not always be equivalent to systemic inflammation, yet it may still represent the only information available to guide clinical decision-making during a patient’s initial assessment.

Attrition was also a concern, with approximately 25% of patients lost to follow-up by week eight, potentially introducing selection bias, as the most severely ill patients may have been underrepresented in later assessments. Despite this, energy and protein intake remained relatively stable over time across the cohort, suggesting persistent intake challenges even among patients with low CRP. This may reflect disease progression, nutritional impact symptoms, or insufficient nutritional interventions. Future studies should consider incorporating direct measurements of inflammatory cytokines such as IL-6 to better understand the mechanistic pathways involved and explore whether patients with the lowest intake and highest CRP had the poorest outcomes [36].

## 6. Conclusions

This study demonstrated that systemic inflammation is independently associated with lower energy and protein intake in patients with advanced cancer, suggesting a potential role in the observed nutritional decline beyond the role of inflammation as a marker of disease severity. These findings underscore the importance of considering inflammation when addressing nutritional challenges in cancer care. Future studies should explore whether addressing both inflammation and nutritional intake may help to limit weight loss and enhance patient outcomes in advanced cancer. Routine monitoring of CRP and other inflammatory markers may help identify patients at risk of poor intake and guide timely interventions. However, its use should be interpreted with caution, as there is currently no interventional evidence demonstrating that CRP-guided approaches improve clinical outcomes. Future research should therefore explore whether targeted anti-inflammatory treatments can positively influence nutritional intake and clinical outcomes. Investigating additional inflammatory markers, such as IL-6 and TNF, may further clarify the interplay between inflammation, appetite regulation, and nutritional decline in cancer cachexia. Establishing causality will require randomized controlled trials or longitudinal studies with repeated biomarker measurements.

## Figures and Tables

**Table 1:** Baseline characteristics of the study population (*n* = 170) stratified by C-reactive protein (CRP) levels (<10 mg/L and >10 mg/L). Continuous variables are presented as mean (SD) or median (IQR), and categorical variables as *n* (%).

Variable	CRP < 10 mg/L (*n* = 83)	CRP > 10 mg/L (*n* = 87)	*p*-Value ^a^
**Age (years, median (IQR))**	65 (17)	65 (13)	0.700
**Gender, male (%)**	44 (53)	56 (64)	0.133
**Living situation,** ***n*****(%)**			
Alone	19 (23)	17 (20)	0.160
With spouse/partner	44 (53)	51 (59)	
With spouse/partner and child(ren)	13 (16)	17 (20)	
With child(ren)	7 (8)	1 (1)	
With other adults	0 (0)	1 (1)	
**Primary tumor site, ** ***n*** **(%)**			
Gastrointestinal	18 (22)	27 (31)	0.020
Prostate cancer	18 (22)	20 (23)	
Breast cancer	26 (31)	9 (10)	
Lung cancer	8 (10)	16 (18)	
Urological cancer	6 (7)	9 (10)	
Other	7 (8)	6 (7)	
**Time since diagnosis (weeks), median (IQR)**	92 (274)	54 (131)	0.598
**Metastases (other than bone), ** ***n*** **(%)**			
Lymph nodes	31 (37)	41 (47)	0.197
Liver	33 (40)	35 (40)	0.950
Lung	25 (30)	30 (34)	0.543
CNS	6 (7)	5 (6)	0.695
Other	19 (23)	27 (31)	0.232
**No extraskeletal metastases, ** ***n*** **(%)**	24 (19)	19 (22)	0.225
**Skeletal region of radiation, ** ***n*** **(%)**			
Spinal column	41 (49)	51 (59)	0.228
Pelvis	41 (49)	35 (40)	0.229
Extremities	10 (12)	6 (7)	0.250
Thorax (excl. spinal column)	4 (5)	4 (5)	0.946
Other	1 (1%)	2 (2%)	0.588
**Prescribed radiation dose, ** ***n*** **(%)**			
8 Gy × 1	16 (19)	24 (28)	0.003
4 Gy × 5	16 (19)	32 (37)	
3 Gy × 10	43 (52)	22 (25)	
Other	8 (10)	9 (10)	
**Systemic treatment last 4 weeks, ** ***n*** **(%)**			
Chemo	22 (27)	20 (24)	0.721
Hormonal	32 (39)	22 (27)	0.098
Other	24 (29)	17 (20)	0.208
**Performance status (Karnofsky), ** ***n*** **(%)**			
0–60	18 (22)	26 (30)	0.222
70–100	65 (78)	61 (70)	
**Patient reported weight loss (%), mean (SD)**	2.4 (9.8)	4.6 (5.9)	0.077
**BMI (kg/m^2^), mean (SD)**	24.6 (4.5)	25.0 (4.6)	0.629
**Sarcopenia ** ***n*** **(%)**	29 (38)	36 (43)	0.743
**Risk of malnutrition,** ***n*** **(%) ^b^**			
Well nourished (Score 0–1)	25 (32)	11 (13%)	0.002
At risk/moderate malnutrition (2–8)	37 (48)	41 (48)	
High risk/severe malnutrition (≥9)	15 (19)	33 (39)	
**Appetite loss,** ***n*** **(%)**			
Not at all	39 (47)	29 (33)	0.321
A little	20 (24)	24 (28)	
Quite a bit	14 (17)	19 (22)	
Very much	10 (12)	15 (17)	

^a^ Frequencies were compared using the chi-square test and continuous variables were compared using linear regression. ^b^ Measured using Patient Generated Subjective Assessment Short Form (PG-SGA SF).

**Table 2:** Energy and protein intake and weight loss over time by the CRP group. All patients with data at ≥1 time point are included. Data are shown as mean (SD).

Variable	*n*	CRP ≤ 10 mg/L	*n*	CRP > 10 mg/L
Energy intake, kcal/kg				
Baseline	83	27.4 (12.7)	87	23.6 (10.0)
Week 3	69	27.4 (11.6)	55	23.9 (12.2)
Week 8	65	29.6 (10.6)	47	25.7 (9.0)
Protein intake, g/kg				
Baseline	83	1.12 (0.61)	87	0.90 (0.45)
Week 3	69	1.09 (0.49)	55	0.87 (0.42)
Week 8	65	1.13 (0.40)	47	1.00 (0.36)
Weight loss (%)				
Baseline	85	0.00 (0.00)	93	0.00 (0.00)
Week 3	76	0.36 (2.85)	70	2.67 (3.32)
Week 8	72	0.63 (4.81)	57	3.85 (4.68)

**Table 3:** Mixed linear model of energy and protein intake.

	Energy Intake (kcal/kg/Day)			Protein Intake (mg/kg/Day)		
	Coeff.	SE	*p*_1_	Coeff.	SE	*p*_1_
CRP > 10	−3.55	1.7	0.038	−247	83	0.003
Age (per 10 yrs)	0.006	0.80	0.994	−30.0	37	0.424
Female	−1.48	2.2	0.510	9.95	94	0.915
Karnofsky ≤ 60	0.35	2.0	0.860	−49.2	75	0.510
Primary tumor type ^a^						
Prostate cancer	−3.90	3.2	0.223	136	137	0.320
Lung cancer	1.31	2.9	0.646	270	146	0.064
GI cancer	−2.14	2.8	0.448	−8.91	110	0.936
Urological cancer	−2.14	3.1	0.489	38.0	127	0.766
Other	−4.15	2.8	0.145	9.87	131	0.940
Current treatment ^b^						
Chemotherapy	3.98	1.6	0.016	97.2	73	0.186
Endocrine therapy	−1.09	2.2	0.616	−132	87	0.130
Other	−0.53	1.6	0.739	−24.5	68	0.717
Study visit						
Week 3	−0.42	1.3	0.753	−45.0	60	0.455
Week 8	2.13	1.8	0.253	−9.40	79	0.905
Study visit/CRP interaction						
3w# > 10	0.74	2.0	0.716	2.65	82	0.974
8w# > 10	0.07	2.3	0.977	102	100	0.311
Constant	29.1	5.5	<0.001	1297	261	<0.001

^a^ Breast cancer is the reference category. ^b^ Categories are not mutually exclusive; the reference category to ‘chemotherapy’ is ‘no chemotherapy’, etc.

## Data Availability

The data presented in this study are available on request and with participants’ consent from the corresponding author due to ethical considerations, including the need for anonymization and the protection of participants’ confidentiality.
